# Accuracy of Smart Scales on Weight and Body Composition: Observational Study

**DOI:** 10.2196/22487

**Published:** 2021-04-30

**Authors:** Justine Frija-Masson, Jimmy Mullaert, Emmanuelle Vidal-Petiot, Nathalie Pons-Kerjean, Martin Flamant, Marie-Pia d'Ortho

**Affiliations:** 1 Physiologie-Explorations Fonctionnelles, Fédération Hospitalo-Universitaire APOLLO (Personalised medicine in chronic cardiovascular, respiratory, renal diseases and organ transplantation) Hôpital Bichat Claude Bernard Assistance Publique Hôpitaux de Paris Paris France; 2 Université de Paris Neurodiderot, Institut national de la santé et de la recherche médicale U 1141 Paris France; 3 Digital Medical Hub Hôpital Bichat Claude Bernard, Assistance Publique Hôpitaux de Paris Paris France; 4 Département d’Epidémiologie, Biostatistiques et Recherche Clinique Hôpital Bichat Claude Bernard Assistance Publique Hôpitaux de Paris Paris France; 5 Université de Paris Infection, antimicrobiens, modélisation, évolution, institut national de la santé et de la recherche médicale Paris France; 6 Université de Paris Institut national de la santé et de la recherche médicale U 1149 Paris France; 7 Pharmacie Hôpital Bichat Claude Bernard Assistance Publique Hôpitaux de Paris Paris France

**Keywords:** smart scales, DEXA, obesity

## Abstract

**Background:**

Smart scales are increasingly used at home by patients to monitor their body weight and body composition, but scale accuracy has not often been documented.

**Objective:**

The goal of the research was to determine the accuracy of 3 commercially available smart scales for weight and body composition compared with dual x-ray absorptiometry (DEXA) as the gold standard.

**Methods:**

We designed a cross-sectional study in consecutive patients evaluated for DEXA in a physiology unit in a tertiary hospital in France. There were no exclusion criteria except patient declining to participate. Patients were weighed with one smart scale immediately after DEXA. Three scales were compared (scale 1: Body Partner [Téfal], scale 2: DietPack [Terraillon], and scale 3: Body Cardio [Nokia Withings]). We determined absolute error between the gold standard values obtained from DEXA and the smart scales for body mass, fat mass, and lean mass.

**Results:**

The sample for analysis included 53, 52, and 48 patients for each of the 3 tested smart scales, respectively. The median absolute error for body weight was 0.3 kg (interquartile range [IQR] –0.1, 0.7), 0 kg (IQR –0.4, 0.3), and 0.25 kg (IQR –0.10, 0.52), respectively. For fat mass, absolute errors were –2.2 kg (IQR –5.8, 1.3), –4.4 kg (IQR –6.6, 0), and –3.7 kg (IQR –8.0, 0.28), respectively. For muscular mass, absolute errors were –2.2 kg (IQR –5.8, 1.3), –4.4 kg (IQR –6.6, 0), and –3.65 kg (IQR –8.03, 0.28), respectively. Factors associated with fat mass measurement error were weight for scales 1 and 2 (*P*=.03 and *P*<.001, respectively), BMI for scales 1 and 2 (*P*=.034 and *P*<.001, respectively), body fat for scale 1 (*P*<.001), and muscular and bone mass for scale 2 (*P*<.001 for both). Factors associated with muscular mass error were weight and BMI for scale 1 (*P*<.001 and *P*=.004, respectively), body fat for scales 1 and 2 (*P*<.001 for both), and muscular and bone mass for scale 2 (*P*<.001 and *P*=.002, respectively).

**Conclusions:**

Smart scales are not accurate for body composition and should not replace DEXA in patient care.

**Trial Registration:**

ClinicalTrials.gov NCT03803098; https://clinicaltrials.gov/ct2/show/NCT03803098

## Introduction

Cellular-connected scales, familiarly known as smart scales, are increasingly used at home for weight follow-up. They have been shown to increase the frequency of self-weighing and weight loss [1,2]. They can be connected to other smart objects, such as motion sensors, and thus may help subjects engage in greater physical activity and better nutritional habits. Most available smart scales combine a classic weight scale with a foot-to-foot impedance meter (FFI) that can estimate body composition (ie, fat mass [FM] and fat-free mass [FFM]) by measuring foot-to-foot impedance at different frequency. Whole body FFM is calculated from a model comprising body impedance, height, weight, and age trained with dual x-ray absorptiometry (DEXA) data [3]. Smart scales are easier to use than medical impedance meters since they do not require a supine position and their electrodes are indefinitely reusable. But the accuracy of smart scales depends on the representativeness of the patient population used to train the model and the model itself. Although some FFIs have been compared with DEXA and to medical impedance meters [3,4], no data are available for smart scale FFIs, and the regression equations used are unknown. The purpose of this cross-sectional study was to assess metrologic accuracy of 3 commercially available smart scales compared with DEXA as the gold standard in a population of adult patients from a tertiary hospital physiology unit.

## Methods

### Patients

Consecutive adult patients evaluated for body composition by DEXA at the Bichat Hospital during the study were eligible. Patients were referred for DEXA because of obesity or a chronic condition that can affect body composition (eg, chronic kidney disease, long-term steroids). All patients were included in the analysis except those who refused to participate or whose weight measured by Lunar iDXA (GE Healthcare) exceeded the maximum weight tolerated by the smart scale (eg, 160 kg for DietPack and 180 kg for Body Partner and Body Cardio). Weight and body composition by DEXA was performed by a trained technician according to current practice on a Lunar iDXA. Three smart scales were assessed (Body Partner [Téfal], DietPack [Terraillon], and Body Cardio [Nokia Withings]) during 3 consecutive periods (ie, only 1 scale was tested per patient for feasibility reasons). Patients were not required to be fasting. DEXA and assessment by the smart scale were performed with the patient wearing a hospital disposable gown and their undergarment. A total of 55 patients were planned for each scale.

### Statistics

Patient characteristics were described as median and interquartile range for quantitative variables and percentages for categorical variables. Absolute and relative errors for each scale with respect to DEXA were reported with median and interquartile range. Bland-Altman representations were used to show systematic bias or trend in measurement error. Univariate linear models were used to estimate and test the association between measurement error and possible associated variables. For this analysis, we reported the slope estimation with its 95% confidence interval and the *P* value corresponding to the Wald statistic. We performed no imputation for missing data. The significance threshold was .05. All analyses were performed using R software version 4.0.2 (R Foundation for Statistical Computing).

### Ethical Aspects

This study is part of the Evaluation of the Metrological Reliability of Connected Objects in the Measurement of Medical Physiological Parameters (EvalExplo) study [NCT03803098]. Ethics approval was obtained from Comité de Protection des Personnes Sud Est VI (approval number AU 1443), and written nonopposition was obtained.

## Results

### Patient Characteristics

The final sample for analysis included 53, 52, and 48 patients for each scale, respectively, after taking into account missing data (eg, smart scale not retrieving data despite several attempts for all but one who was excluded because of excessive weight for the scales). Patient characteristics are presented in Table 1. There were no significant differences between the 3 groups.

**Table 1:** Description of included patients.

Characteristic		Scale 1: Body Partner (n=53)	Scale 2: DietPack (n=52)	Scale 3: Body Cardio (n=48)
Sex, male, n (%)		17 (32)	27 (52)	32 (67)
Age in years, median (IQR^a^)		46 (37-58)	48 (40-56)	51 (43-62)
Weight (kg), median (IQR)		83.4 (71.4-102.1)	80.5 (69.2-99.3)	82.2 (75-94)
Height (cm), median (IQR)		166 (162-173)	169.5 (164-175)	166 (159-173)
**BMI (kg/m^2^), median (IQR)**		30.3 (25.0-37.6)	28.4 (24.8-34.1)	31.2 (26.1-35.5)
	BMI < 25, n (%)	13 (25)	14 (27)	9 (19)
	25 ≤ BMI < 30, n (%)	11 (21)	16 (31)	13 (27)
	30 ≤ BMI < 35, n (%)	14 (26)	11 (21)	12 (25)
	BMI ≥ 35, n (%)	15 (28)	11 (21)	14 (29)
Body fat (kg), median (IQR)		33.5 (21.5-49.8)	31.2 (23.5-43.4)	28.5 (16.0-41.4)
Muscular mass (kg), median (IQR)		49.1 (42.0-56.1)	52.0 (43.8-57.1)	47.6 (42.8-53.8)
Bone mass (kg), median (IQR)		2.77 (2.23-3.02)	2.74 (2.33-3.21)	2.56 (2.17-2.93)
Excess mass (kg), median (IQR)		1.04 (0.84-1.29)	0.98 (0.81-1.27)	4.8 (2.2-6.2)

^a^IQR: interquartile range.

### Accuracy of the Scales

All 3 scales gave rather accurate weights, with a median absolute difference of less than a kilogram compared with DEXA. The median absolute error for body weight was 0.3 kg (interquartile range [IQR] –0.1, 0.7), 0 kg (IQR –0.4, 0.3), and 0.25 kg (IQR –0.1, 0.5) for Body Partner (scale 1), DietPack (scale 2), and Body Cardio (scale 3), respectively.

For fat mass, absolute errors were –2.2 kg (IQR –5.8, 1.3), –4.4 kg (IQR –6.6, 0), and –3.7 kg (IQR –8.0, 0.3), respectively. Body fat was globally underestimated in all 3 scales. For muscular mass, absolute errors were 4.50 kg (IQR 0.4, 7.3), –6.6 kg (IQR –9.4, –3.6), and 4.0 kg (IQR 0.1, 7.6), respectively. Muscular mass was thus underestimated by scale 2 and overestimated by scales 1 and 3.

**Figure 1:**
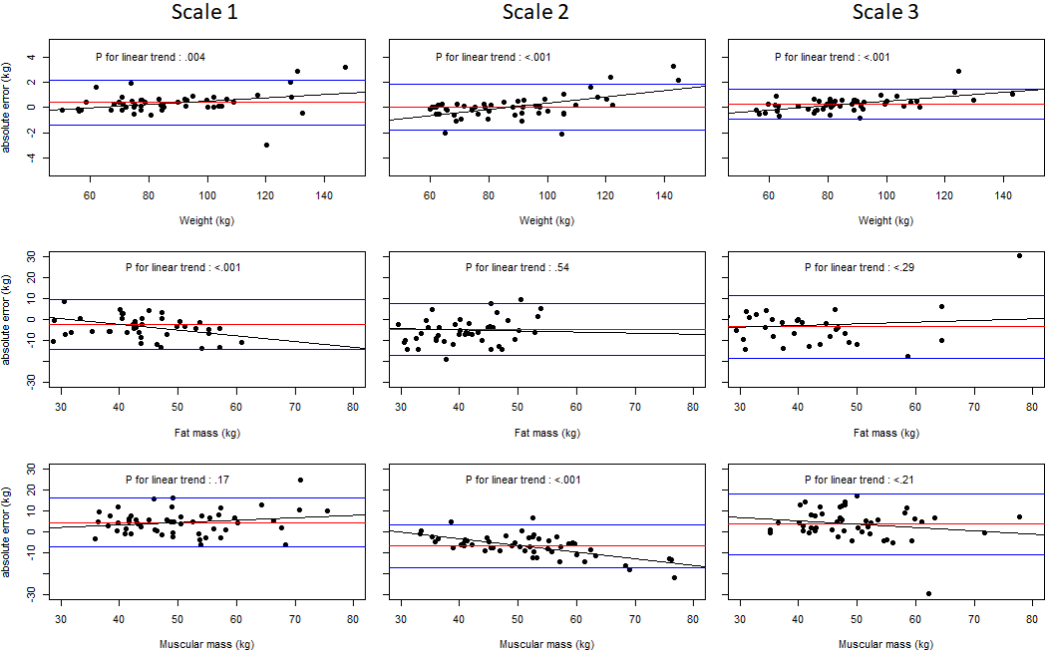
Bland-Altman plots of the three scales for body weight, fat mass, and muscular mass. The red line indicates mean error, blue lines indicate 2.5 and 97.5 percentiles of the error distribution, and the black line represents a linear fit.

Bland-Altman graphs are presented in Figure 1 for weight, body fat, and muscular mass for the 3 scales. They show a significant linear trend of body weight on measured weight, but absolute errors remain reasonable and compatible with clinical practice. However, significant linear trends for fat and muscular mass (scales 1 and 2, respectively) lead to very high errors.

### Factors Associated With Measurement Error

Tables 2 and 3 show the factors associated with fat and muscular mass measurement error. Factors associated with fat mass measurement error were weight for scales 1 and 2 (*P*=.03 and *P*<.001, respectively), BMI for scales 1 and 2 (*P*=.034 and *P*<.001, respectively), body fat for scale 1 (*P*<.001), and muscular and bone mass for scale 2 (*P*<.001 and *P*<.001, respectively). Factors associated with muscular mass error were weight and BMI for scale 1 (*P*<.001 and *P*=.004, respectively), body fat for scales 1 and 2 (*P*<.001 and *P*<.001, respectively), and muscular and bone mass for scale 2 (*P*<.001 and *P*=.002, respectively).

We found no factor associated with measurement error of fat or muscular mass for scale 3. Sex did not show a significant influence on measurement error.

**Table 2:** Factors associated with fat mass measurement error for the 3 scales (univariate linear regressions).

Characteristic		Scale 1: Body Partner (n=53)				Scale 2: DietPack (n=52)				Scale 3: Body Cardio (n=48)		
		Estimate	95% CI	*P* value	Estimate		95% CI	*P* value	Estimate		95% CI	*P* value
**Sex**		—^a^	—	.96	—		—	.24	—		—	.57
	Female	1 (ref)	—	—	1 (ref)		—	—	1 (ref)		—	—
	Male	0.095	–3.5 to 3.7	—	2.1		–1.4 to 5.7	—	1.3		–3.4 to 6.0	—
Age in years		0.023	–0.091 to 0.14	.69	0.042		–0.088 to 0.17	.52	–0.064		–0.25 to 0.12	.49
Weight (kg)		–0.065	–0.12 to –0.0	.03	0.18		0.11 to 0.24	<.001	0.079		–0.032 to 0.190	.16
Height (cm)		0.024	–0.15 to 0.20	.79	0.054		–0.17 to 0.28	.64	0.2		–0.035 to 0.43	.09
**BMI (kg/m²)**		—	—	.03	—		—	<.001	—		—	.83
	BMI<25	1 (ref)	—	—	1 (ref)		—	—	1 (ref)		—	—
	25<BMI<30	2	–2.8 to 6.7	—	1.2		–2.8 to 5.2	—	0.97		–5.8 to –8.4	—
	30<BMI<35	–2.8	–7.2 to 1.6	—	3.8		–0.55 to 8.2	—	–1.5		–8.4 to 5.4	—
	BMI>35	–4.4	–8.7 to –0.013	—	9.8		5.42 to 14.2	—	–1.4		–8.0 to 5.3	—
Body fat (kg)		–0.27	–0.40 to –0.14	<.001	–0.053		–0.23 to 0.12	.54	0.08		–0.07 to 0.23	.29
Muscular mass (kg)		0.0081	–0.16 to 0.17	.92	0.42		0.29 to 0.54	<.001	0.16		–0.084 to 0.41	.19
Bone mass (kg)		–0.45	–3.4 to 2.5	.76	6.2		3.2 to 9.3	<.001	2.4		–1.6 to 6.3	.24

^a^Not applicable.

**Table 3:** Factors associated with muscular mass measurement error for the 3 scales (univariate linear regressions).

Characteristic			Scale 1: Body Partner (n=53)							Scale 2: DietPack (n=52)							Scale 3: Body Cardio (n=48)				
			Estimate		95% CI		*P* value		Estimate			95% CI		*P* value		Estimate			95% CI		*P* value
**Sex**			—^a^		—		.52		—			—		.07		—			—		.47
	Male	1 (ref)		—		—		1 (ref)			—		—		1 (ref)			—			
	Female	1.1		–2.4 to 4.7		—		–2.6			–5.47 to 0.26		—		–1.7			–6.3 to 2.9			
Age in years			–0.039		–0.150 to 0.072		.49		0.098			–0.0054 to 0.20		.06		0.073			–0.11 to 0.25		.42
Weight (kg)			0.11		0.054 to 0.16		<.001		–0.048			–0.12 to 0.02		.16		–0.068			–0.18 to 0.042		.22
Height (cm)			0.085		–0.087 to 0.26		.33		–0.15			–0.331 to 0.032		.10		–0.2			–0.43 to 0.025		.08
**BMI (kg/m²)**			—		—		.003		—			—		.87		—			—		.77
	BMI<25	1 (ref)		—		—		1 (ref)			—		—		1 (ref)			—		—	
	25<BMI<30	–1.9		–6.3 to 2.5		—		–0.43			–4.4 to 3.5		—		–0.41			–7.0 to 6.2		—	
	30<BMI<35	1.9		–2.2 to 6.0		—		–1.67			–6.0 to 2.7		—		2			–4.7 to 8.7		—	
	BMI>35	5.8		1.8 to 9.9		—		–1.11			–5.5 to 3.2		—		2.14			–4.4 to 8.7		—	
Body fat (kg)			0.28		0.15 to 0.40		<.001		0.23			0.10 to 0.36		<.001		–0.063			–0.21 to 0.085		.40
Muscular mass (kg)			0.11		–0.047 to 0.27		.17		–0.32			–0.42 to –0.21		<.001		–0.15			–0.40 to 0.089		.21
Bone mass (kg)			2.2		–0.64 to 4.94		.13		–4.2			–6.9 to –1.6		.002		–2.1			–6.0 to 1.9		.30

^a^Not applicable.

## Discussion

### Principal Findings

To our knowledge, this is the first study to assess metrologic accuracy of commercially available smart scales (scale 1: Body Partner, scale 2: DietPack, scale 3: Body Cardio). We show that all scales were reasonably accurate for body weight but not body composition. Total body weight was associated with fat mass and muscular measurement error for scales 1 and 2, but we were not able to find factors associated with error measurement for scale 3.

### Possible Explanations for the Lack of Accuracy

Smart scales combine a classic weight scale and an FFI, which has been widely available for almost three decades and has been compared with medical impedance meters and DEXA in several publications [5-8]. They have been shown to be more sensitive to differences in morphology than whole body impedance measurements, since their data depend upon an extrapolation of measurements made on the lower part of the body to the entire body. Indeed, Bousbiat et al [3] conducted an extensive technical study on FFIs. They found that measurement can be affected by surface contact (ie, foot size and width) and sweat but also by foot position on the scale and flexion of the legs. Surface contact with the electrodes will vary depending on the subject’s foot length and width, and thus can be affected by total body weight and total height. Since there is no precise guidance on the scales for subjects on where to put their feet during body composition estimation, this may partly explain the differences between DEXA and scales. In clinical settings, clinicians or technicians should pay attention to the subject’s position during measurement. In the same way, subjects should be advised not to bend their legs. At home, subjects should try to follow directions given by the scale as closely as possible and keep the same position on the scale for follow-up.

In FFIs, whole body FFM is calculated from a regression equation expressing resistance generally as a function of height, weight, and age determined by comparison with DEXA data, while FM is calculated as the weight of FFM. Each smart scale has its own regression equation. It is thus plausible that BMI can affect error measurement. We did not find any factor associated with error measurement for scale 3 despite a very high dispersion, which can have different explanations: our study may be underpowered to detect such association or unobserved variables not part of the secret regression implemented in the smart scale may explain the residual error.

### Clinical Relevance of These Data

Weight was accurately measured by all 3 scales, but body fat was underestimated. For scales 1 and 2, we found a significant effect of higher body weight on fat mass error; this error remains small compared with total body weight in patients with obesity but can be of importance in patients with normal or underweight. Ross et al [9] compared the weight from in-person visits and BodyTrace brand smart scales in 58 patients and found a mean bias of 1.1 (SD 0.8) kg, 95% CI 0.5 to 2.6, but agreement seemed lower for patients weighing above 110 kg.

It is unlikely that body composition will be followed by DEXA in the clinical setting, since DEXA uses x-rays. Since smart scales are widely available, it is possible that patients will follow their body composition at home. Thus, if the first body composition is assessed by DEXA, clinicians and patients should be aware that there might be a difference in body composition, which can reach 1 kg. Also, follow-up in the clinical setting should use the same connected device to ensure repeatability of measurements.

### Interest for Follow-Up

Despite very poor accuracy for estimating body composition, some authors reported potential interests of in-home use of smart scales. Indeed, in several randomized studies, when compared with commercial weight management programs or standard weight loss counseling, smart scale use was found to allow a greater proportion of participants to achieve significant weight loss after several months (3 to 12 months) [1,2,10]. In these studies, no data were available on body composition and its evolution. Also, several studies report a greater weight loss in patients using smart scales than in patients self-reporting weight loss or being weighted only during visits [11,12]. Last, data exist on the importance of weight variability in final weight loss and weight maintenance [13-15]; using smart scales at home with automated data treatment could help health care professionals and patients in achieving and maintaining greater weight loss [16].

### Strengths and Limitations

To our knowledge, this is the first cross-sectional study assessing metrologic accuracy of commercially available smart scales. We compared these scales to DEXA, which is considered the gold standard for body composition assessment.

Included patients were evaluated for body composition either because of obesity or because of a chronic condition (steroids, chronic renal failure, etc) without obesity. Thus, our sample covers a wide range of body weight: underweight, lean, and obese patients. The setting of the study in a tertiary hospital explains the relatively small proportion of patients with obesity since the main reason for prescribing DEXA was long-term treatment with steroids. A specific study focusing on patients with obesity and extreme obesity would be required since they are likely to benefit the most from body composition evaluation and follow-up and since the error on fat mass is affected by total weight. This should be done keeping in mind the maximum weight supported by the scales (180 kg for Body Cardio and Body Partner and 160 kg for DietPack), while DEXA supports higher weight (230 kg on our machine).

Finally, although body composition estimation is quick with the smart scales, total experimental time was 15 to 30 minutes for each scale due to scale and app setup and patient information. Thus, it was not possible to evaluate the 3 scales on the same patient or replicate measures. However, due to the very poor accuracy of these scales shown in this study, comparisons between scales and estimation of intraindividual variability would have limited relevance. This study included only a limited number of patients. However, while it is always possible that the studied scales would perform better on an independent sample, the study results are so clear that it is also unlikely that a higher sample size could change our conclusion relative to the accuracy of body composition estimation.

### Conclusion

Our study shows that although smart scales are accurate for total body weight, they should not be used routinely to assess body composition, especially in patients with severe obesity. Further studies are needed to clarify their utility in patient follow-up.

## Abbreviations

DEXA: dual x-ray absorptiometry
FFI: foot-to-foot impedance meter
FFM: fat-free mass
FM: fat mass
IQR: interquartile range
